# The Safer Prescription of Opioids Tool (SPOT): A Novel Clinical Decision Support Digital Health Platform for Opioid Conversion in Palliative and End of Life Care—A Single-Centre Pilot Study

**DOI:** 10.3390/ijerph16111926

**Published:** 2019-05-31

**Authors:** Roger Flint, Deans Buchanan, Scott Jamieson, Alfred Cuschieri, Shady Botros, Joanna Forbes, Jacob George

**Affiliations:** 1 Medical School, University of Dundee, Ninewells Hospital and Medical School, Dundee DD1 9SY, UK; rflint@nhs.net (R.F.); a.cuschieri@dundee.ac.uk (A.C.); joanna.forbes@nhs.net (J.F.); 2 NHS Tayside Ninewells Hospital, Dundee DD1 9SY, UK; deansbuchanan@nhs.net (D.B.); scottjamieson@nhs.net (S.J.); sbotros@nhs.net (S.B.)

**Keywords:** Clinical Decision Support Systems, Opioid Analgesics, Focus Groups, Pilot Projects, Palliative Care

## Abstract

Opioid errors are a leading cause of patient harm. Active failures in opioid dose conversion can contribute to error. Conversion is complex and is currently performed manually using tables of approximate equivalence. Apps that offer opioid dose double-checking are available but there are concerns about their accuracy and clinical validation. This study evaluated a novel opioid dose conversion app, The Safer Prescription of Opioids Tool (SPOT), a CE-marked Class I medical device, as a clinician decision support (CDS) platform. This single-centre prospective clinical utility pilot study followed a mixed methods design. Prescribers completed an initial survey exploring their current opioid prescribing practice. Thereafter prescribers used SPOT for opioid dosage conversions in parallel to their usual clinical practice, then evaluated SPOT through a survey and focus group. SPOT matched the Gold Standard result in 258 of 268 (96.3%) calculations. The 10 instances (3.7%) when SPOT did not match were due to a rounding error. Users had a statistically significant increase in confidence in prescribing opioids after using SPOT. Focus group feedback highlighted benefits in Quality Improvement and Safety when using SPOT. SPOT is a safe, reliable and validated CDS that has potential to reduce harms from opioid dosing errors.

## 1. Introduction

Active failures such as slips, lapses and mistakes are inevitable in opioid delivery, and are the major contributing factor to opioid errors in palliative care inpatient services [1]. One aspect prone to active failure is opioid dose conversion [2]. There is wide variability in prescriber competence in performing these calculations. This is a source of prescribing error, including over- and under-dosing, and it may be a risk factor for overdose death [3]. A retrospective review of in-patient palliative care services identified that opioid errors account for 32% of all reported medication errors, and that 84% of reported opioid errors reached the patient [4]. Smartphone apps are available to support clinicians in performing opioid dose conversion, but there are concerns about the reliability, accuracy and validity of these tools [5].

The Safer Prescription of Opioids Tool (SPOT) is a Class I Conformité Européenne (CE) -marked medical device, designed to allow clinicians to validate their mathematical opioid conversions safely and quickly. It was built in Javascript using HTML components, and is available at www.opioidcalculator.co.uk [6].

SPOT has inbuilt warnings to caution prescribers to consider dose adjustments in impaired renal function. SPOT is a clinician decision support (CDS) tool. SPOT was developed in accordance with the Scottish Palliative Care Guidelines’ (SPCG) advice on opioid prescribing [7]. SPOT does not account for patient factors that may affect equianalgesic opioid conversions, including tolerance, opioid sequencing, frailty or liver function. SPOT is not a substitute for clinical acumen and does not include the conversion of methadone due its unique pharmacodynamics.

The aim of this study was to evaluate the clinical utility of SPOT as a CDS platform in opioid dose conversion using clinical data across primary, secondary and tertiary care settings. The secondary objective was to use the novel data collected by SPOT to describe the opioid conversion prescribing landscape regarding frequency and use of the opioids within SPOT during the study period.

## 2. Materials and Methods 

This clinical utility study followed a mixed methods design. The study population included all male and female patients in primary, secondary and tertiary care settings undergoing opioid switch in the Palliative Care, Oncology and Primary Care settings in NHS Tayside, Scotland, during the study period. The data collection period for the clinical study was 5 months (1 May–30 September 2017). We excluded patients with acute pain presentations, defined as short-term pain associated with surgery or traumatic injury, chronic pain presentations not in the last 12 months of life and those aged <16 years old. The prescriber sample comprised of doctors and non-medical prescribers (NMPs) with a license to prescribe opioids, collectively termed “end users” working in the above settings, and consisted of all of those approached that agreed to participate in the study. End users received a login to the SPOT website, a copy of SPOT’s instructions for use (Supplementary Material 1), and a link to an instructional video on YouTube (Supplementary Material 2).

All end users were asked to complete a pre-intervention qualitative questionnaire to explore their current prescribing practice (Appendix A). During the study, end users were requested to perform all opioid dose conversions using their current existing practice method, and thereafter repeat the calculation using SPOT. Participants were made aware that the responsibility of the final dosing decision on prescription lay with them, and that SPOT was to be used only as a research tool. On conclusion of the data gathering period, users were asked to participate in a survey of their confidence in prescribing opioids (Appendix B), and comment on the usability of SPOT by completing a modified system usability score (SUS) questionnaire [8]. 

SPOT recorded all conversion data and non-patient-identifiable demographics in a cloud-based database. It automatically and remotely collected all necessary data on the use of the CDS. A Trial Steering Committee oversaw the study. On conclusion of the study period, all end users were invited to a focus group. Users were asked to take part in an online email survey of their confidence in prescribing opioids to gauge whether they felt SPOT made a difference to their confidence after performing an equianalgesic opioid conversion.

Ethical opinion was sought and waived for this study (EOSRES Ref: 2015PP01).

## 3. Results

### 3.1. Quantitative Results

#### 3.1.1. Conversions Performed

A total of 210 drug-to-drug conversions were recorded over the study period and 76 breakthrough calculations recorded. Of the conversions, the majority (62%) were for opioid conversion due to cancer pain. Some survey respondents completed multiple calculations, others did not complete any calculations.

The opioids used during the study (Table 1) correlated with the data collected in the pre-study questionnaire (Appendix A). Morphine and oxycodone were respectively the first and second-most frequently used opioids. The most common index opioid was morphine, and the most common target opioid was oxycodone. 

#### 3.1.2. Conversion Accuracy

Of the 286 calculations, 18 drug-to-drug calculations did not have equianalgesic equivalents for the chosen preparations in the latest iteration of the Scottish Palliative Care Guidelines [7]. These calculations were removed from the final analysis, leaving a total of 192 drug-to-drug calculations and 76 breakthrough calculations. 

SPOT matched the SPCG in 258/268 calculations (96.3%). The 10 non-matches were due to SPOT’s algorithm for the handling of fentanyl patches. If a calculated result exactly bordered two patch strengths, the error resulted in SPOT rounding-up to the higher-strength patch, instead of rounding-down to the lower strength patch. This was corrected and independently validated in a subsequent version of SPOT. Excluding fentanyl patch dose conversion, SPOT correctly matched SPCG dose conversions 100% of the time.

### 3.2. Qualitative Results

#### 3.2.1. Pre-SPOT Use Survey Questionnaire Results

All eligible prescribers were invited to complete an initial survey before using SPOT (Table 2). At baseline, only 75% of initial questionnaire respondents always double-checked their opioid conversions. The most popular method for double-check was a colleague or consultant. Despite this, 52% of respondents admitted to ever having a situation whereby they had amended a calculation, and 35% had an incidence when they stated that they were unable to perform a calculation without assistance. Of the pre-study users, 7% were not aware that opioid doses or choices were influenced by renal function. Prescribing resources used by the study population included the SPCG (94%), and pharmacy colleagues (62%). The second-most popular resource was “own knowledge”, with 63% of respondents being colleagues that double-checked themselves with their own knowledge and methods. The categories are not mutually exclusive. Almost all users (98%) felt it beneficial to their practice and for patient safety to have an easy way to double-check their calculations. Two-thirds of respondents felt that using a CDS to calculate a drug dosage would save time versus a manual double-checking calculation. Some respondents subsequently did not use the tool over the study period. Other participants used the tool frequently and performed numerous calculations.

#### 3.2.2. Post-SPOT Use Survey Questionnaire Results

**Figure 1:**
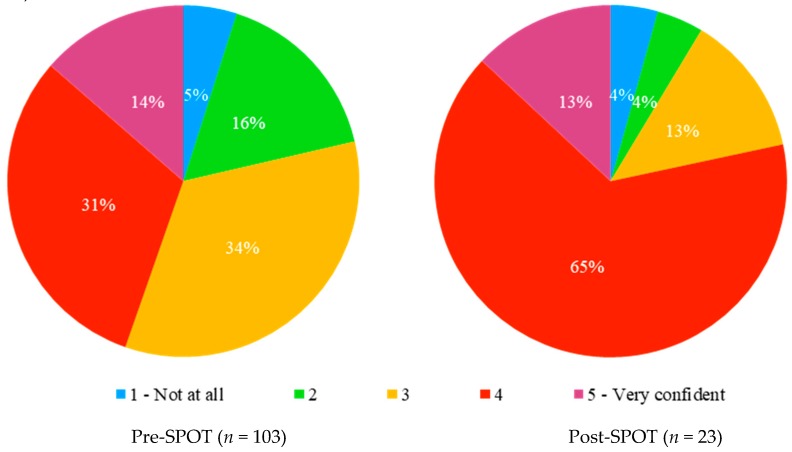
Users’ self-reported confidence with opioid conversions, before and after the SPOT study.

Confidence in prescribing opioids was borderline significantly higher in the post-study group than in the pre-study group (Figure 1) (Cochran–Armitage test for trend, Z = −1.9266, *p*-value = 0.05403). 

The post-use survey questionnaires are detailed in Appendix B. Themes identified for not using the CDS over the study period included a lack of time to double-check conversions, and no formal requirement to double-check calculations during the study period. Note that all respondents that used the CDS felt that the double-checking feature was useful. Two-thirds of respondents felt that using a CDS to calculate a drug dosage would save time versus a manual double-checking calculation. All users considered it important that the CDS was available on their smartphone. 

#### 3.2.3. SPOT System Usability Scale Assessment

The system usability scale was used to assess the usability of SPOT; SUS score = 72.7 (*n* = 18). A score of over 68 is above average usability. 

#### 3.2.4. SPOT Post Study Focus Group

Three of the most frequent end users of the tool participated in the focus group in a claims, concerns and issues format. The key themes identified are detailed in Table 3. 

The focus group data (*n* = 3) (Table 3) reflect concerns about the strength of fentanyl patches used in the CDS, the conversion factor to fentanyl patches, and questioned ways to improve safety of the CDS’s usability. The end users that participated in the focus group felt that the tool was useful as an educational resource, and had safety and quality improvement benefits. Usability was both a claim and a concern, reflecting the contrast between a visually friendly tool and one that required developer improvement for use on different platforms. There are opportunities for CDS, including as an educational resource, a quality improvement mechanism and a safety tool. 

Of the respondents to the pre-study questionnaire, 23% stated that they converted opioids on a daily basis. However, only 210 calculations were collected over the six-month study period. The focus group survey identified a number of reasons why prescribers did not engage with the CDS. Common themes included a perceived lack of need for the tool due to the simplicity of an opioid conversion task, frequency of performing opioid conversions and comments on the usability of the research version of the tool. 

Of those that did engage with SPOT, common themes included using SPOT as a “double-checking device” and as a safety check as identified by the focus group that highlighted reassurance as a benefit of SPOT. 

## 4. Discussion

The intention of this study was to quantitatively and qualitatively evaluate the clinical utility of a novel opioid dose conversion CDS in a real-world pilot study. The information gathered provides insight into the challenges of using technology for complex opioid conversions. Almost all of those participating in the survey would double-check their calculations if there was a simple, quick and safe option to do so. There is a time cost associated with the most common current practice of seeking help from colleagues; colleagues may be remote, or busy with other tasks, which may delay the double-check of the dosage, and potentially impact on the patient awaiting analgesia. SPOT performed well in terms of dose conversion and system usability tests. The issue with dose rounding and fentanyl has been addressed in the second version of SPOT. Data were not collected regarding the number of patients in the last days of life, and methadone was not included in SPOT as it is not part of the SPCG formulary for generalist users. 

Clear cautions were raised when performing opioid rotations; that it is not simply a mathematical conversion and that prescribers must prescribe according to their clinical knowledge. This study did not determine whether an increase in prescriber confidence led to a reduction in opioid prescribing error. Our initial survey identified low confidence and variable competence in performing equianalgesic opioid conversions. The second-most commonly cited resource, “own knowledge”, likely reflects that the participants who volunteered to participate in the study had an interest in palliative medicine. The lack of variety of opioids prescribed over the study period may reflect a repertoire of drugs that prescribers are familiar in using, and that patients may not have the benefit of a wider range of drug choices due to an individual prescriber’s confidence.

We found variable adherence to guidelines. For example, despite SPCG guidance to the contrary, not all of the respondents altered their choice of index opioid despite a reduced estimated glomerular filtration rate (eGFR).

There are a number of limitations to this study. SPOT was piloted in only one Health Board; however, we recruited prescribers from all grades and skill levels to ensure we captured all potential end users of SPOT. The response rate was lower for the post-use survey, which may limit the generalisability of the post-use feedback. We do not know if those that completed the first questionnaire completed the second survey, and vice-versa. One potential explanation of the attrition is that the study period encompassed six months, which included the summer holidays and August change-over of junior doctors. Many of those doctors moved out of the study area and were lost to follow up. Of the remaining balance, a number of users did not engage with the post-SPOT survey. 

The study was not funded to reimburse travel or locum cover for participants to attend the focus group. Therefore, we engaged with three of the most frequent users to participate in this activity in order to understand concerns and feedback on the CDS. The focus group participants may not be a representative sample of all of the views of the participants in the study. Participation in the focus group may have been improved by using other methods such as telephone or video conferencing. Future research is required to assess the use of CDS. This should include wide-scale feasibility testing in multiple boards and a cluster randomised study to investigate whether SPOT influences opioid prescription error. Given the novel data gathered by SPOT, there is the possibility to use it to refine the equianalgesic dosage conversion factors in different patient populations, bringing the potential for personalised equianalgesic dosage conversion.

## 5. Conclusions

This study evaluated the use of SPOT in clinical practice using contemporaneous clinical data. SPOT was found to improve end user self-reported confidence when performing opioid dose conversion in palliative and end-of-life care.

## Figures and Tables

**Table 1:** Opioids used during the study period, recorded as the starting (index) and the resulting opioid (target) of the opioid switch.

Opioid	As Index Opioid (*n*)	As Target Opioid (*n*)
Alfentanil	26	41
Buprenorphine	2	4
Codeine	16	3
Diamorphine	6	7
Dihydrocodeine	1	1
Hydromorphone	10	9
Fentanyl	0	29
Morphine	81	53
Oxycodone	68	63

**Table 2:** Breakdown of pre-study questionnaire respondents by role and area of practice.

Respondents	*n*	Area of Practice	*n*
Junior Doctor	35	Primary Care	38
Nurse	19	Secondary Care	59
Consultant	7	Tertiary Care	6
General Practitioner	29		
Pharmacist	6		
Non-Training Grade Doctor	7		

**Table 3:** Focus group feedback—claims, concerns and issues key themes.

Claims	Concerns	Issues
Educational resource	Usability	Improving safety of use
Quality improvement	Formulary	Improving app usability
Safety	Accuracy of conversion	Revising the app formulary
Double-checking		
Ease of use		
Evidence		

## Supplementary Materials

The following are available online at https://www.mdpi.com/1660-4601/16/11/1926/s1, Supplementary Material 1: Instructions for use document, Supplementary Material 2: SPOT YouTube video link.

## Appendix A

Pre-SPOT Questionnaires.

**How confident do you feel with opioids conversions? (1–5 scale)**	**Very (5)**	**4**	**3**	**2**	**Not at all (1)**	
If eGFR is reduced (<30) does this usually alter your choice of opioid?	Yes	No				
What resources do you use when making drug conversions	Choice:	Own Knowledge	Scottish Palliative Care Guidelines	Colleague	Senior Colleague	Other source, please specify
Do you always double-check your equianalgesic conversions	Yes	No				
If yes, how?	Choice	Colleague	Consultant	Senior Colleague	Pharmacist	Other
If not, why not?	Choice	Confident with prescribing	No time	Availability of colleague	Other	
If you could easily double-check your calculations, would you?	Yes	No				
Have you ever had a situation where you had to amend a calculation?	Yes	No				
Have you ever had a situation where you were unable to perform a calculation?	Yes	No				

## Appendix B

Post-SPOT Questionnaire.

**Following this trial, how confident do you feel with opioids conversions? (1–5 scale)**	**Very (5)**	**4**	**3**	**2**	**Not at all (1)**	
If eGFR is reduced (<30) does this usually alter your choice of opioid?	Yes	No				
What resources do you use when making drug conversions?	Choice:	Own Knowledge	Scottish Palliative Care Guidelines	Colleague	Senior Colleague	Other source, please specify
Do you always double-check your conversions?	Yes	No				
If yes, how?	Choice	Colleague	Consultant	Senior Colleague	Pharmacist	Other
If not, why not?	Choice	Confident with prescribing	No time	Availability of colleague		
If you could easily double-check your calculations, would you?	Yes	No				
Have you ever had a situation where you had to amend a calculation?	Yes	No				
Could SPOT resolve this situation?	Yes	No				
Have you ever had a situation where you were unable to perform a calculation?	Yes	No				
Could SPOT resolve this situation?	Yes	No				
Are you familiar with the opioid conversion calculator, SPOT?	Yes	No				
Have you used the opioid conversion calculator, SPOT?	Yes	No				
If you did not use the tool, what was the reason?	Choice					
