# Supplementary material for: The Safer Prescription of Opioids Tool (SPOT): A Novel Clinical Decision Support Digital Health Platform for Opioid Conversion in Palliative and End of Life Care—A Single-Centre Pilot Study
Source: Int J Environ Res Public Health. 2019 May 31;16(11):1926. doi: 10.3390/ijerph16111926 (PMC6612362; doi:10.3390/ijerph16111926)
Supplement: Supplementary file 1 [file ijerph-16-01926-s001.zip › ijerph-493995 Suppmentary Materials/ijerph-493995 Suppmentary 1.pdf]

SPOT

The Safer Prescription of Opioids Tool

Instructions For Use

April 2017

Roger Flint

Contents:

1. Introduction, Technology, Background
2. Accessing SPOT - First time Usage
3. Accessing SPOT - Subsequent and Ongoing Usage
4. Accessing SPOT - Forgot Password
5. The Home Screen
6. New Opioid Conversion
7. Previous Conversions
8. Syringe Driver Compatibility and Palliative Care Guidelines
9. Table of Equivalence and Patches
10. About and Feedback
11. Logout

Chapter 1: Introduction:

SPOT is a new kind of medical device. It is a 'Clinician decision support' tool (CDS).

SPOT is designed to educate and aid you in your prescribing practice.

SPOT does not replace your clinical judgement.

The aim of SPOT is to help prescribers when prescribing opioids in Palliative and End of Life Care situations.

It does this by:

- Linking directly to the Scottish Palliative Care Guidelines
- Sends you a copy of your equianalgesic dose calculation results
- Lists the available preparations in the pharmacy for your selected drug
- Calculates the equianalgesic conversion between two opioids.
- Saving a list of your calculations for revalidation purposes.

SPOT is based on what I needed as a junior prescriber. It has been reviewed by senior consultants in Palliative care across Scotland, including Dr Deans Buchanan.

I hope that you find SPOT a useful resource. Please email me if there is anything that you feel should be improved: rflint@nhs.net.

Deans Buchanan, Jacob George, Roger Flint. February 2017

Technology:

SPOT is a ‘web app’.

A ‘web app’ or ‘Website application’ is software that runs on a website. It looks like an ‘app’ that you download to your smartphone, but allows you to access it using different platforms such as Windows computers, smartphones and Apple macs.

SPOT is CE marked. It is a Class I Medical Device, and is registered with the Medicines and Healthcare Regulatory Authority in the UK.

Background:

SPOT was developed by a small team of doctors, app developers and a regulatory expert, and has been funded by grants from Scottish Enterprise and PATCH, and personal contribution.

Chapter 2: Accessing SPOT - First Time Usage:

IMPORTANT - PLEASE NOTE

Not all NHS computers are compatible with SPOT due to the age of their software.
If SPOT does not work on your NHS computer, please try your smartphone, tablet or a University Computer.

First time usage - Step 1: Email invitation

When granted access to SPOT, you will receive the following email to your NHS address:

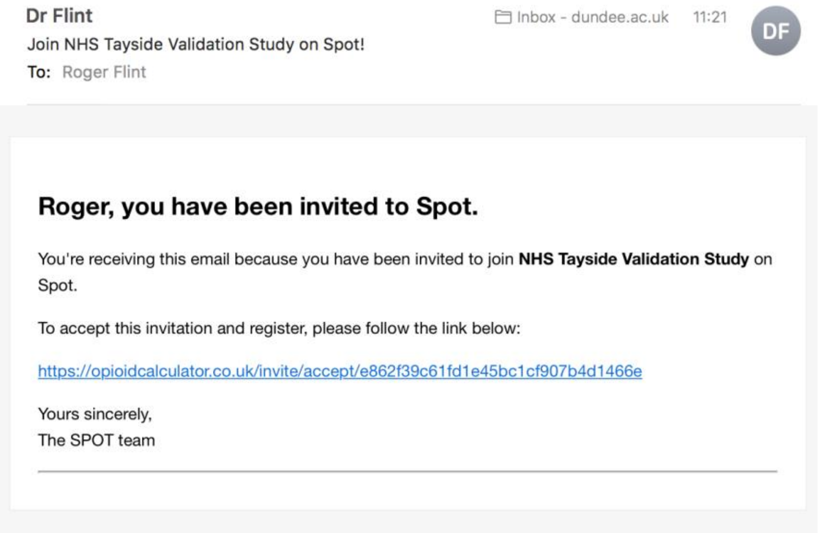

Dr Flint Inbox - dundee.ac.uk 11:21 DF

Join NHS Tayside Validation Study on Spot!

To: Roger Flint

Roger, you have been invited to Spot.

You're receiving this email because you have been invited to join **NHS Tayside Validation Study** on Spot.

To accept this invitation and register, please follow the link below:

https://opioidcalculator.co.uk/invite/accept/e862f39c61fd1e45bc1cf907b4d1466e

Yours sincerely,
The SPOT team

First time usage - Step 2: Choosing a password

On a compatible computer, or on your smartphone, Click the highlighted link in the email to be taken to the registration page. You will be asked to chose a password to access SPOT:

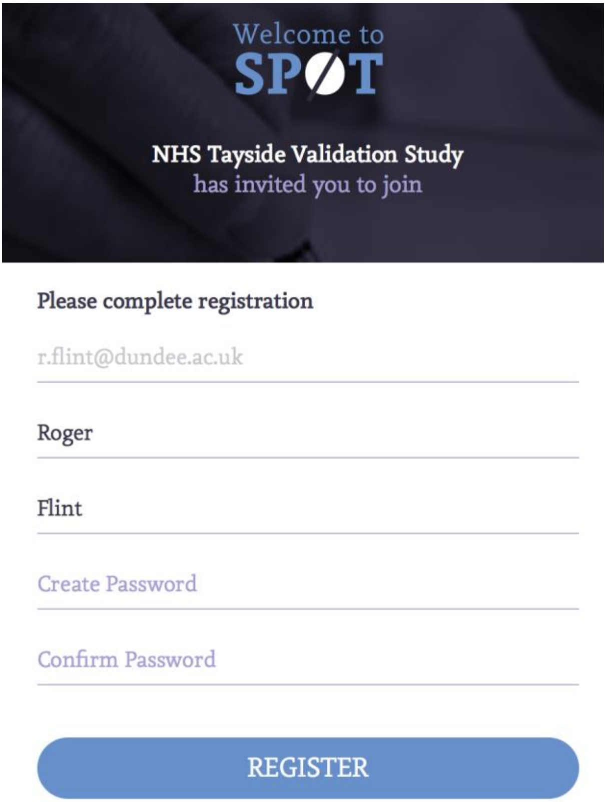

Once you enter a password above and click register, SPOT will save your password. Please keep a note of your password.

Click the ‘Back to Login’ button to login and start using SPOT.

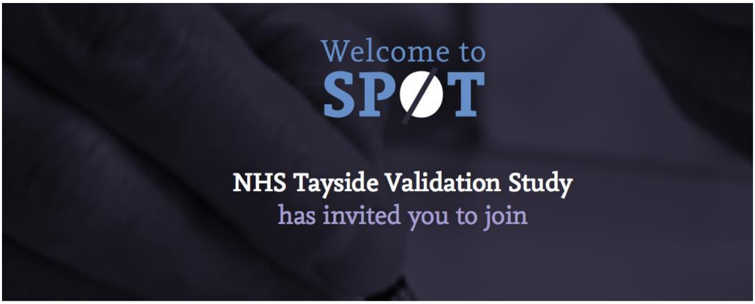

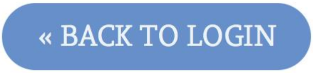

Move to next chapter.

Chapter 3: Accessing SPOT - Subsequent and ongoing use

IMPORTANT - PLEASE NOTE

Not all NHS computers are compatible with SPOT due to the age of their software.
If SPOT does not work on your NHS computer, please try your smartphone or a University Computer.

Please read 'Accessing SPOT - First usage' If you have not already done so

Step 1: Login page

Once your account is set up, go to:
www.opioidcalculator.co.uk

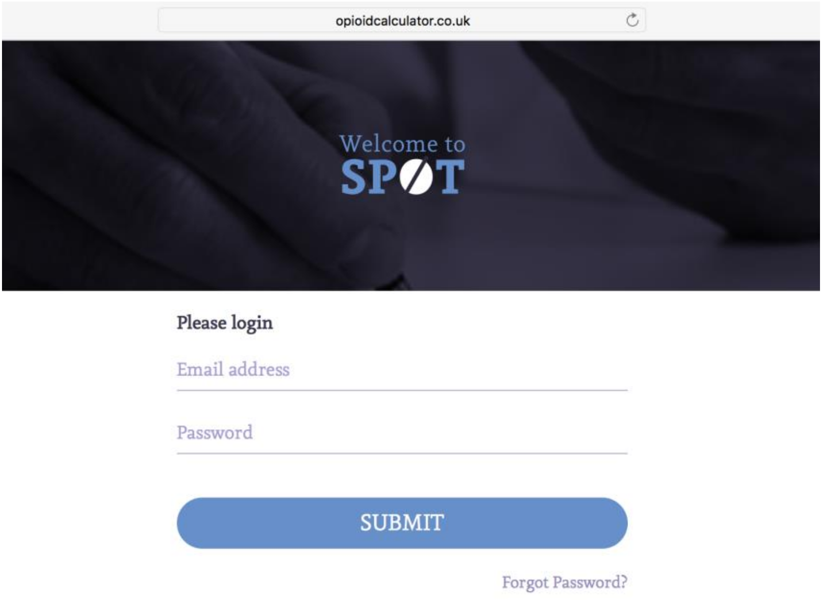

If using a compatible internet browser, you will be presented with the login page:
If presented with a light-purple blank page, the internet browser you are using is not compatible. Please try using a different device.

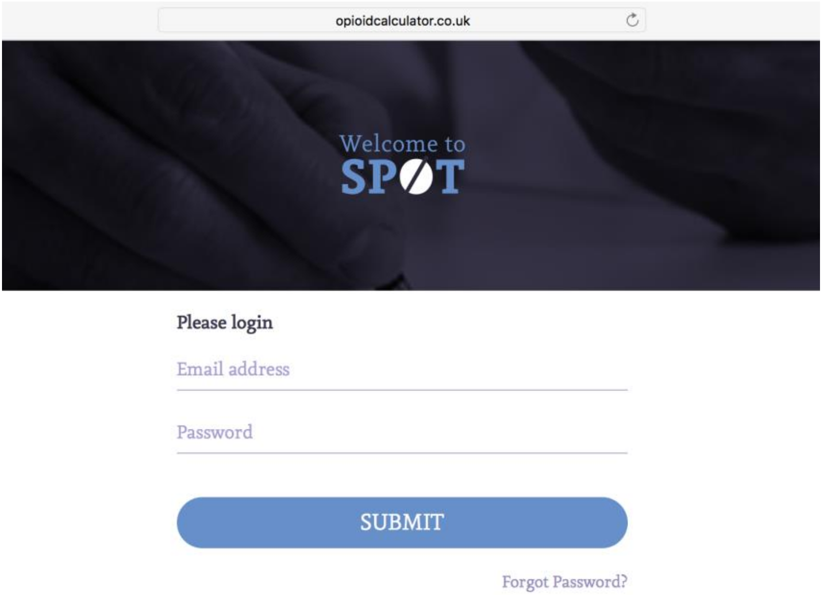

Step 2: Start using SPOT.
In the ‘Email Address’ section, please enter your NHS email address.

Under ‘Password’, enter the password you chose on initial setup.

Click ‘Submit’ to login to SPOT.

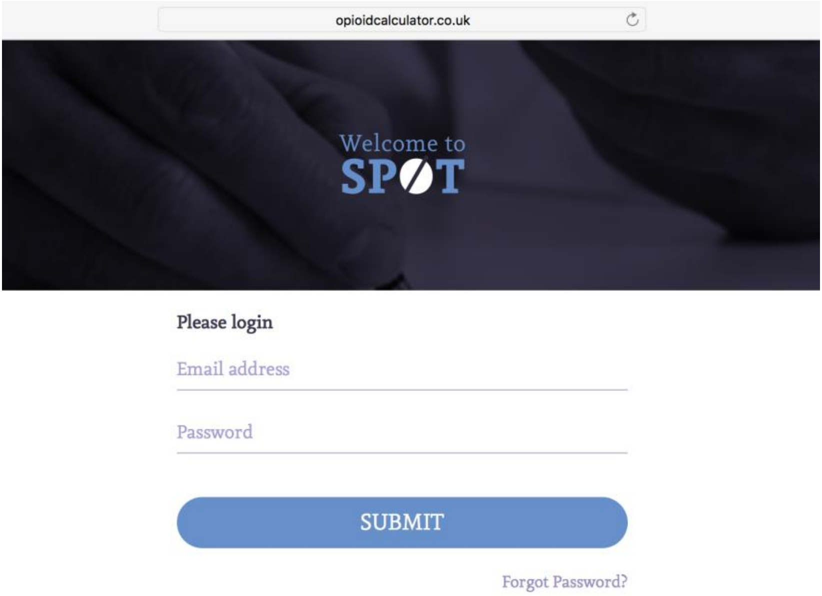

Chapter 4: Forgotten Password

If you forget your password, click the ‘Forgot Password’ link on the login page:

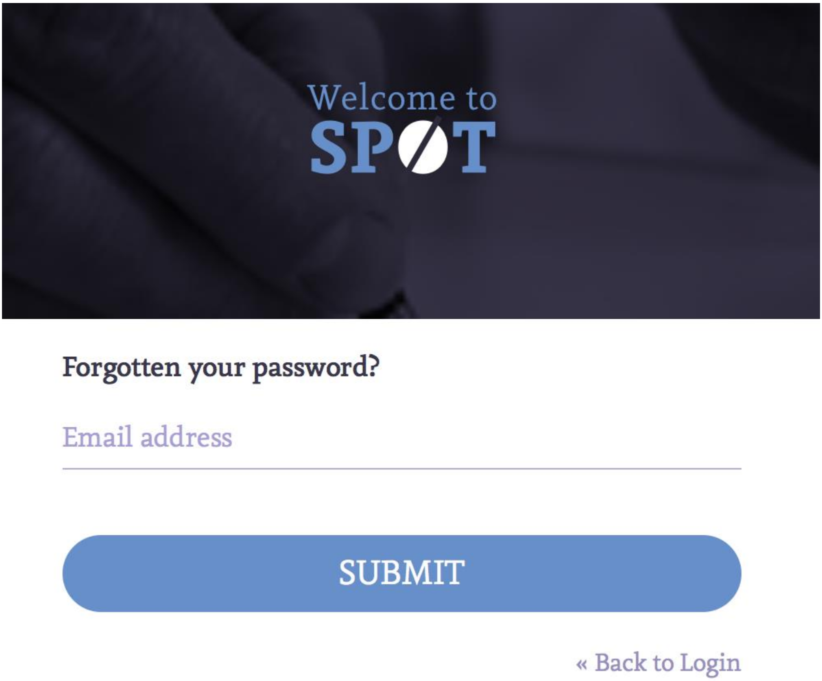

SPOT will ask you to enter your NHS email address.

Do so, and click ‘Submit’

An email link with instructions of how to reset your password will be sent to your NHS email address.

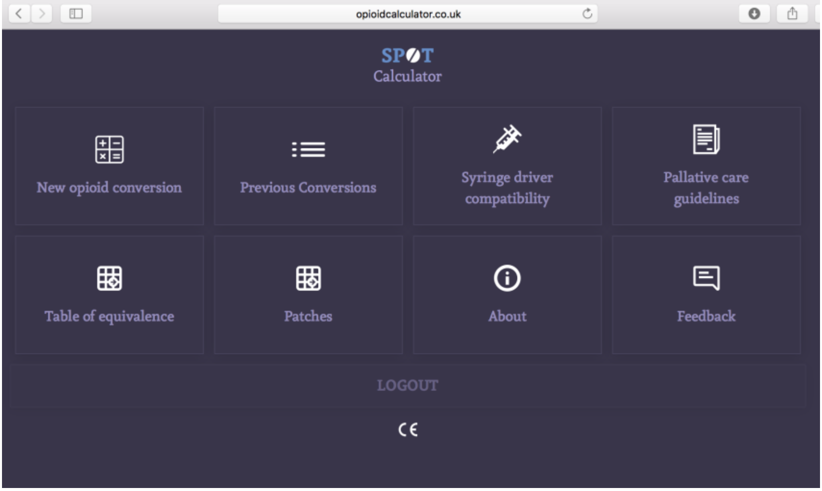

Chapter 5: SPOT - The Home Screen:

Once you successfully login to SPOT, you will see the 'Home Screen"

The 'Home Screen' provides links to SPOT's functionality.

These are as follows:
1. New Opioid Conversion
2. Previous Conversions
3. Syringe Driver Compatibility
4. Palliative Care Guidelines
5. Table of Equivalence
6. Patches

7. About
8. Feedback

Additionally, the ‘Home Screen’ Contains the ‘Logout” button, and the CE designation.

Chapter 6: New Opioid Conversion

Important Note:

SPOT is a clinician decision support application. It is not a prescribing device.

You are encouraged to use SPOT to double check your own calculations, but you CANNOT use SPOT to prescribe.

You MUST calculate your own drug conversions before using SPOT.

You are ultimately responsible for your prescriptions.

New Opioid Conversion - Step 1:

To access the equianalgesic converter, click on ‘New

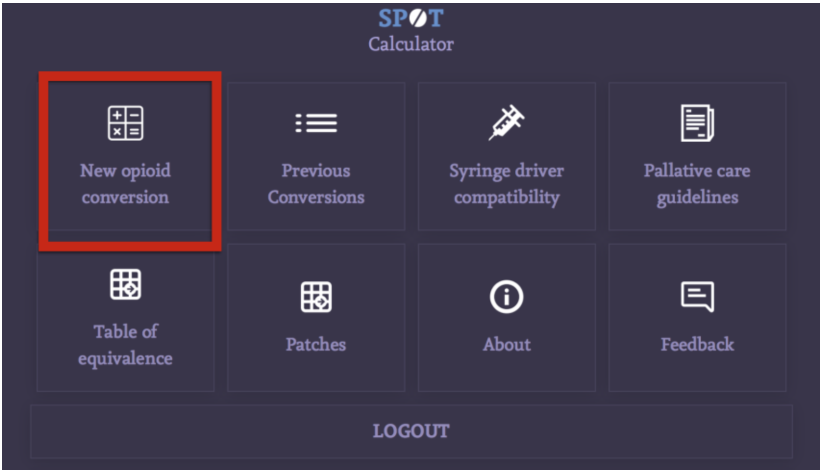

Opioid Conversion’ on the ‘Home Screen’:

New Opioid Conversion - Step 2:

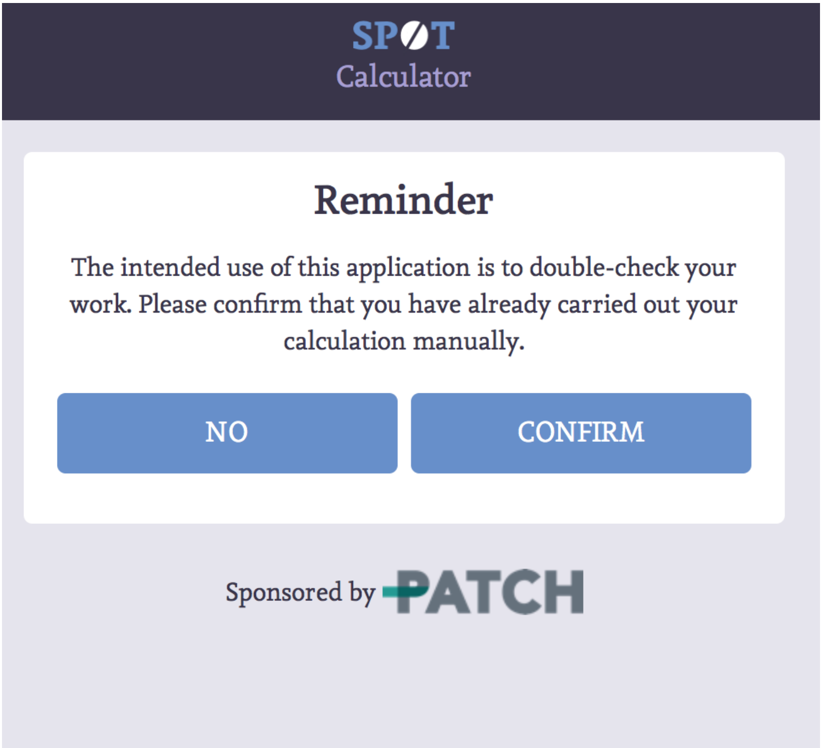

The following page appears:
As noted above, you must perform your own calculation first.
Once you have performed your own dose conversion, click ‘Confirm’ to continue. The following page will load.

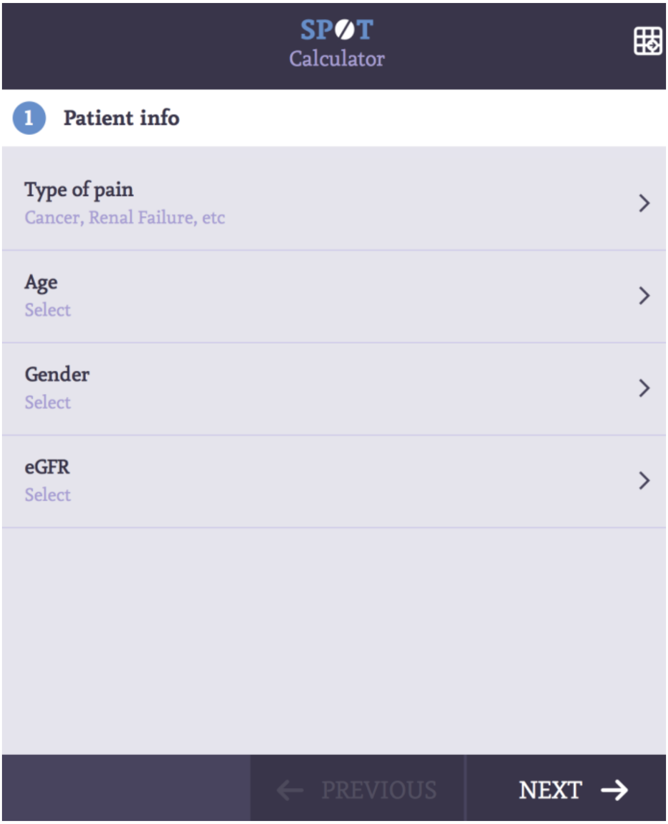

New Opioid Conversion - Step 3: Patient information.

This page asks for information about your patient. The requested information is **not** patient identifiable. However, it should allow **you** to identify the patients that you have seen that day. These information form the basis of the information of patients that you have seen, and must be accurate.

Once complete, click ‘Next’

Click on each heading to input the requested patient information.

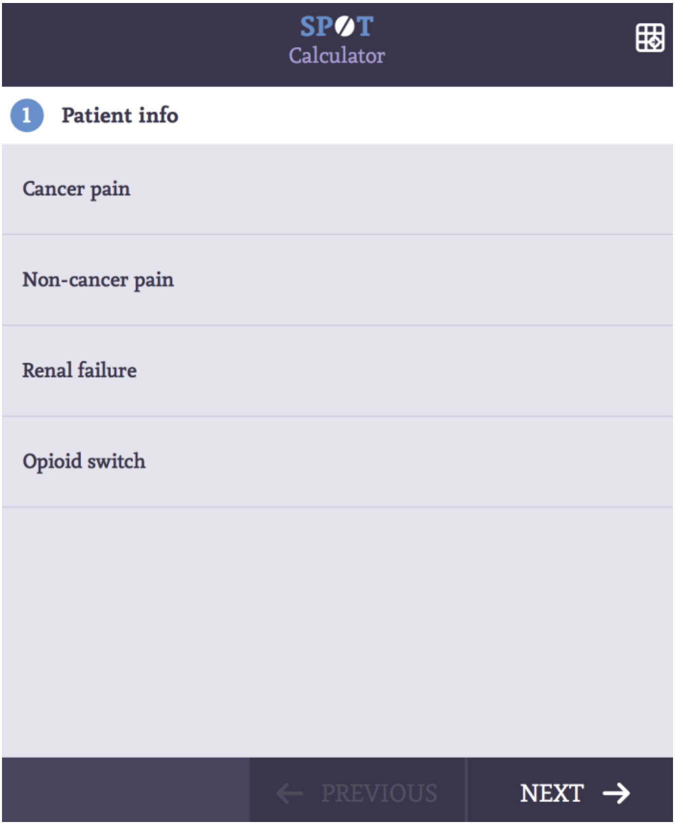

New Opioid Conversion - Step 4: Reason for use:
This page asks you to input the reason that you are using SPOT. Please chose the most accurate heading. If none are applicable, choose ‘Opioid Switch’.
Once complete, click “Next’

New Opioid Conversion - Step 5+6: Age and Gender.

Likewise, enter the patient's Age and Gender by clicking on the heading and using the keyboard to input the correct figures. You will be left with something similar to the

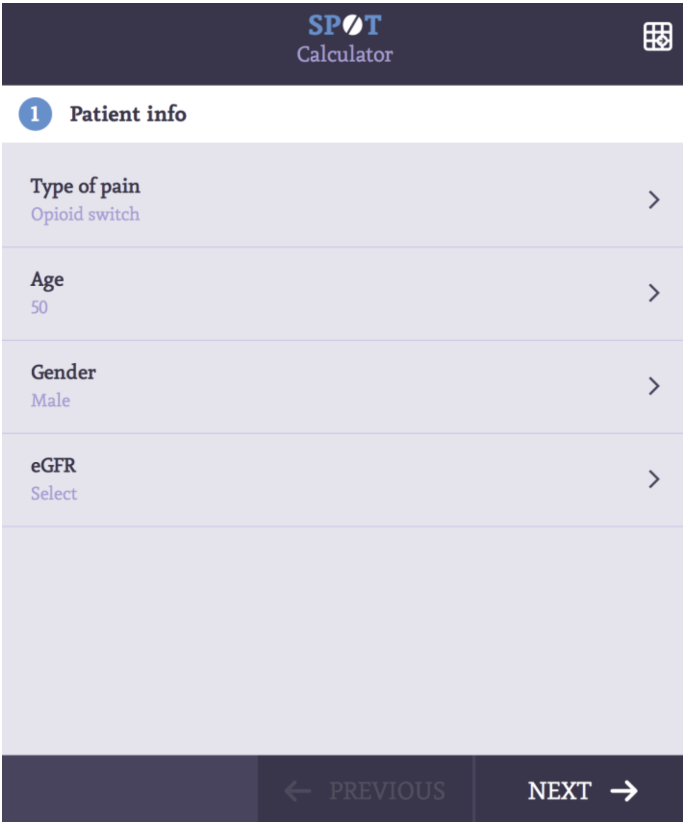

below:

New Opioid Conversion - Step 7: eGFR

The next stage is to enter the patient’s eGFR. This is a measure of renal function, and affects clearance of certain opioids. As discussed previously, SPOT is a clinician decision support device, NOT a prescribing device. As such it DOES NOT correct for eGFR. You must do any

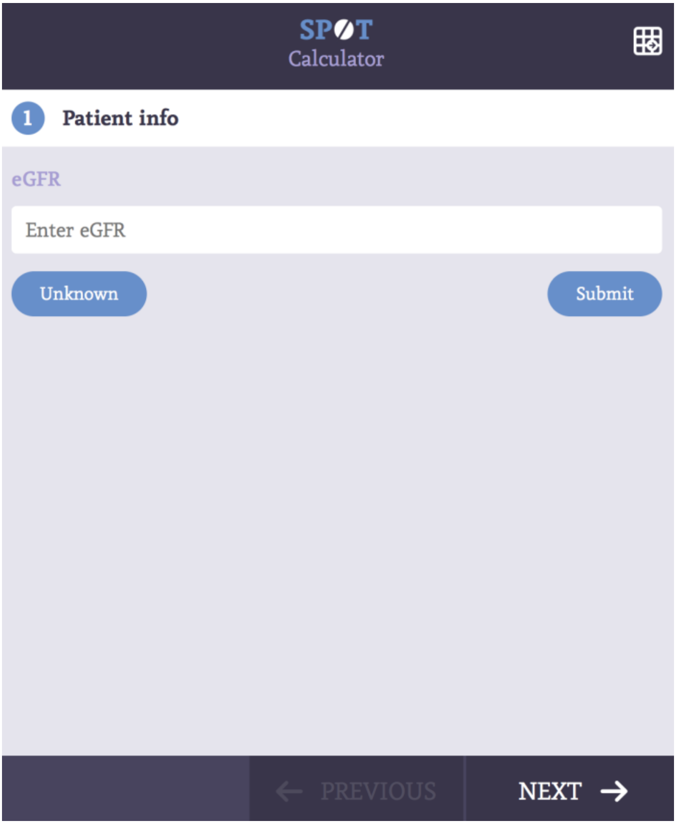

such correction manually.

If you know the patient's eGFR, use the keyboard to enter above, and then click 'Submit'. If you do not know, use the 'Unknown' button, and click 'Next'.

New Opioid Conversion - Step 8: eGFR warning and next page

If you select an eGFR <30, or an unknown eGFR, SPOT

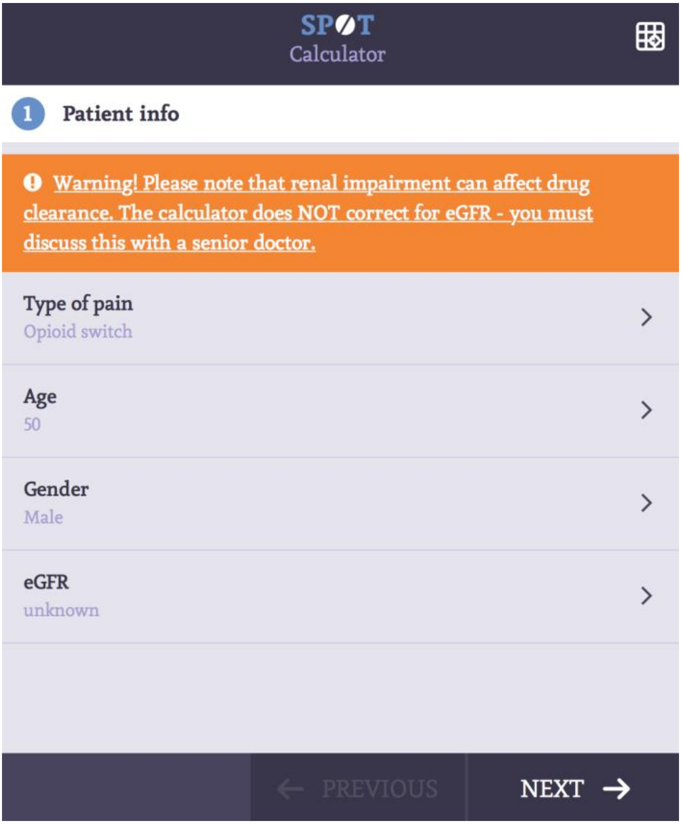

will warn you.

Click ‘Next’ to acknowledge the warning and continue.

New Opioid Conversion - Step 9: Patient Current Opioid

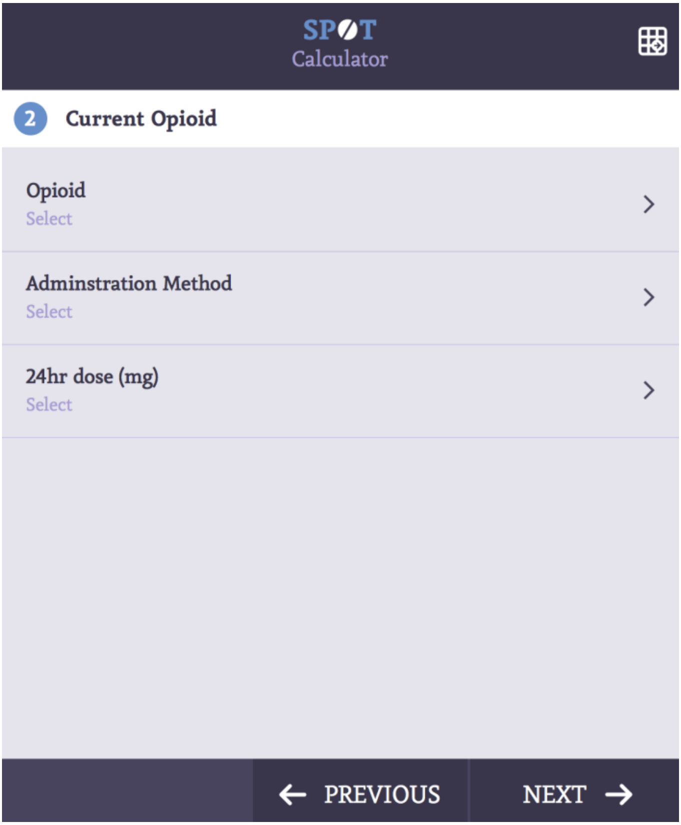

Details:
This page requires you to enter your patient’s current opioid regimen.

Please select ‘Opioid’, and chose the correct opioid from the drop-down list.

Then select ‘Administration Method’ and you will be given a list of administration methods relevant for your selected drug. Choose one of these routes.

Finally, select 24 hour dose. You are required to input the total 24 hour dosage of the opioid selected above via the selected route of administration.

IMPORTANT NOTE: to avoid confusion, dosages are re-quested in **mg** ONLY.

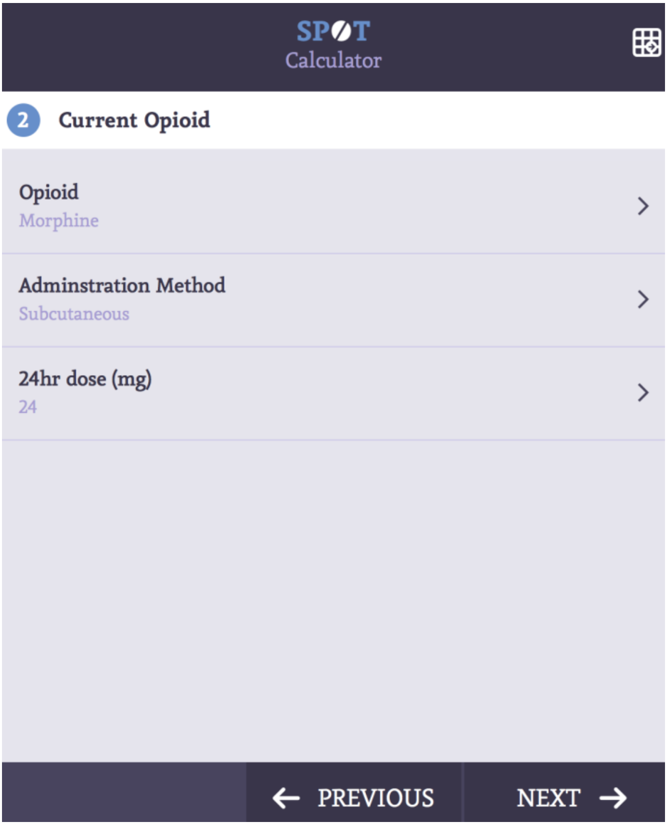

You should end up with something similar to the below:
Example: Patient’s last total 24 hour dosage was 24mg of subcutaneous morphine.

Once the selected Opioid, Administration Method and 24 Hour dose (mg) are correct, click ‘Submit’ to move to the next page.

New Opioid Conversion - Step 10: Patient New Opioid

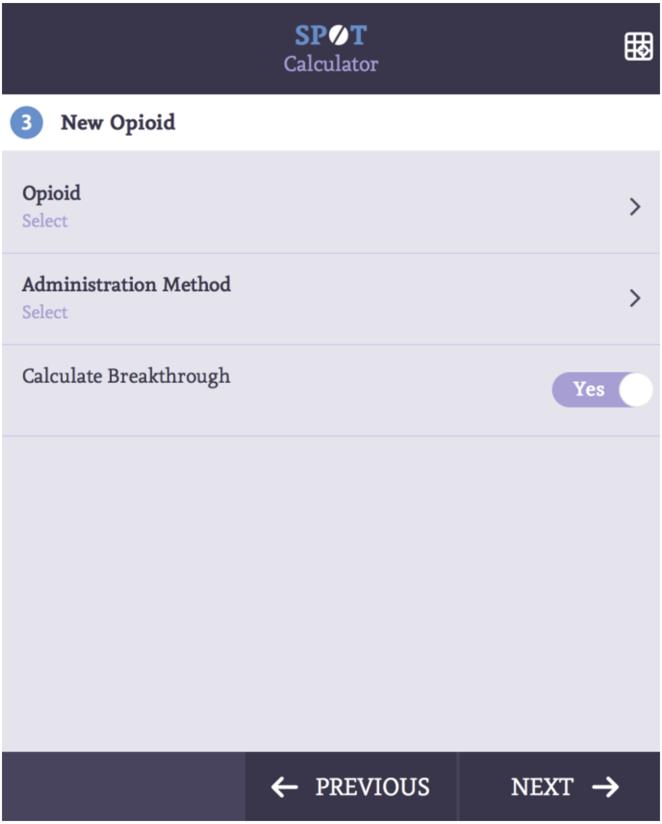

Details:

This page requests details of the opioid that you wish to switch your patient to. Please enter the Opioid and Administration Method as per the previous screen.

Should you wish to calculate a ‘Breakthrough’ dosage, select ‘Yes’ on the slide button.

IMPORTANT NOTE:
Breakthrough dosage calculations use the Scottish Palliative Care Guidelines convention of 1/6th of the total 24

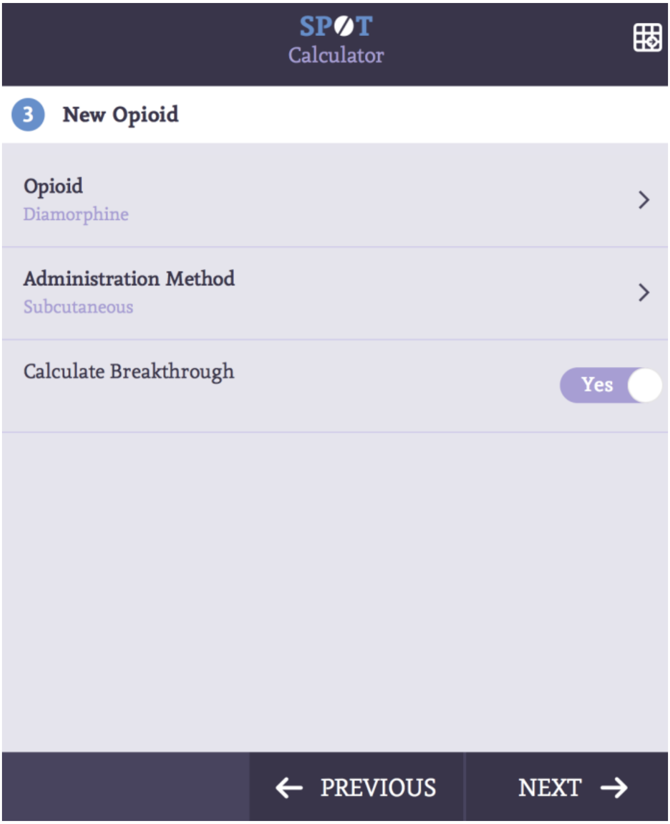

hour dose as the breakthrough dosage.

Example: New opioid chosen of Subcutaneous Diamorphine and requesting SPOT calculate a breakthrough:

Once you have selected your Opioid and Administration route, and chosen whether to you wish SPOT to calculate a breakthrough or not, click ‘Next’ to proceed to the next page.

New Opioid Conversion - Step 11: **Research version only**

Step 11 asks you, the user, to record the answer to your own calculations. This is for the validation study, and will

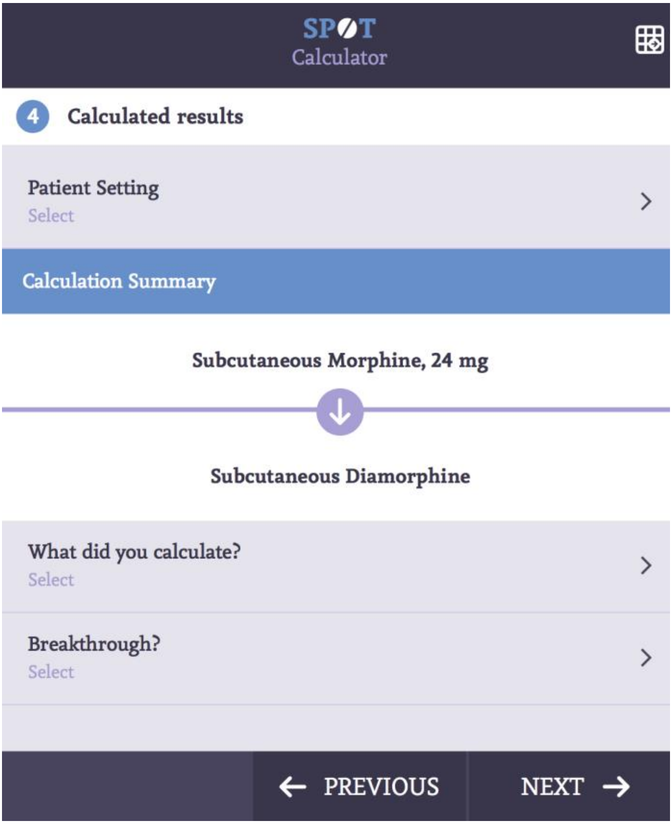

not be present following validation.

This is so that we can validate SPOT using clinical data, comparing the result that you have calculated compared to that which SPOT produces.

This page requests three inputs:

1. The patient setting. I.e. where you saw the patient.

Options are as follows:

- Community Out Patient
- In Patient GP Hospital
- In Patient Acute Hospital
- In Patient SPCU
- Other

2. The result of the conversion that you calculated for the total 24 hour dosage ‘Calculation result’.

3. Your breakthrough calculation result.

Once you have inputed the answers to your own calculations and chosen the setting, click ‘Next’ to continue.

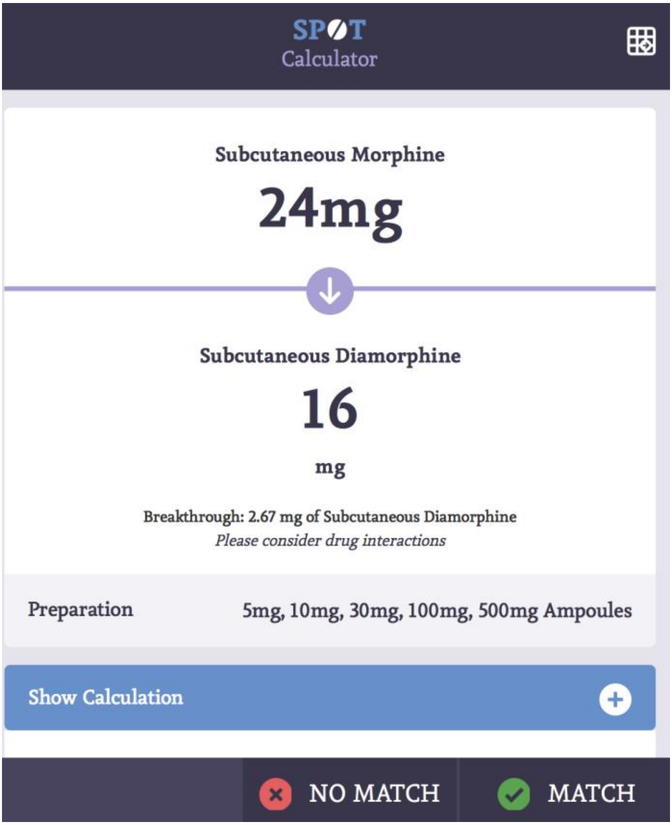

New Opioid Conversion - Step 12: Results.

The next page shows SPOT's calculation results, the preparations available for the selected drug in the pharmacy, the breakthrough calculation, and gives the option to view the calculation pathway that SPOT used.

Should you wish to view the calculation pathway, select

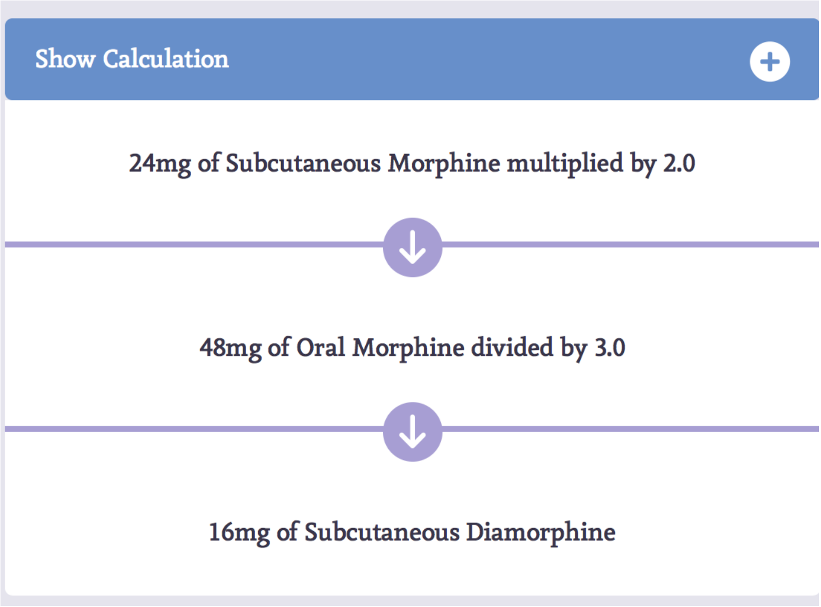

‘Show Calculation’

Finally to complete the calculation, SPOT presents you

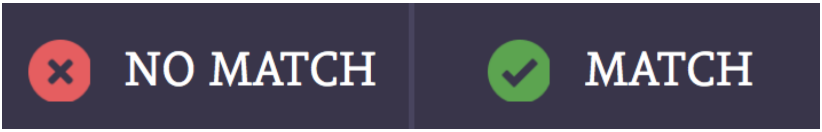

with ‘Match’ and ‘No Match’ options.
If your manual calculation is the same as SPOT’s calculation, select the ‘Match option’.

If your manual calculation is not the same as SPOT's calculated answer, select the 'No Match' option.
New Opioid Conversion - Step 13: Match / No Match:

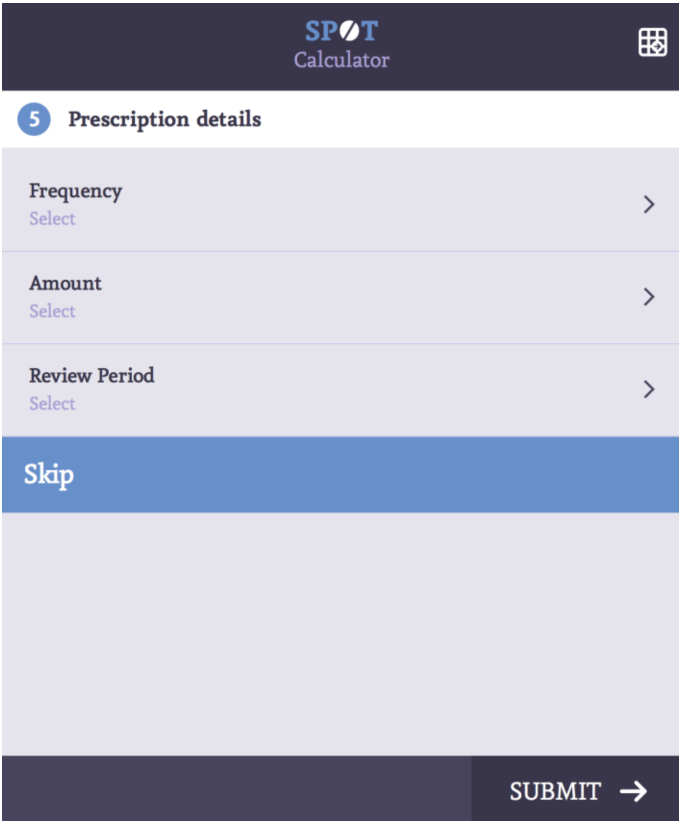

Option 1 - Match:

If your manual calculation answer is the same as SPOT's calculated answer, choose the 'Match option"

SPOT will ask you for the details of the prescription that you wrote for the patient.

These details will be emailed to you. Once the prescription details have been entered, select ‘Submit’ to save the calculation and return to the ‘Home Screen’.

Should you not wish to record your prescription details, select ‘Skip’. This will save the calculation, but not your prescription details, and take you back to the ‘Home Screen’.

Option 2 - No Match

Select ‘No Match’ If your manual calculation answer is

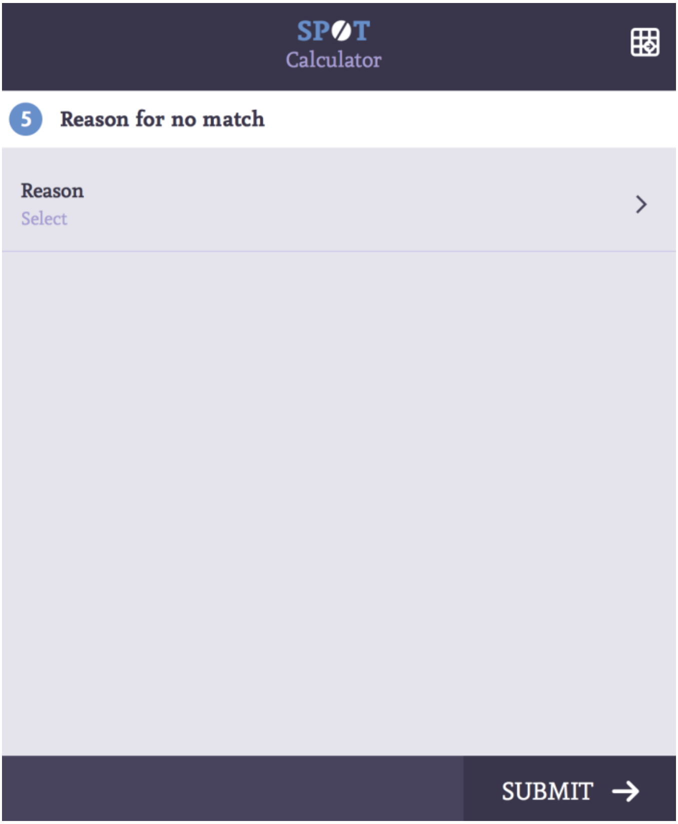

not the same as SPOT’s answer.

SPOT will ask you to fill out the reason for ‘No Match’

The reasons available are as follows:

- Manual miscalculation
- Low eGFR
- Clinical decision
- Preparation of drug unavailable
- Dosage of drug not physically possible
- Other
- The app is incorrect

If you do have a ‘No Match’, and select ‘Other’ please leave a comment as to why SPOT did not match.

Once you select a reason, SPOT will return you to the ‘Home Screen’.

Chapter 7: Previous Opioid Conversions:

SPOT is designed to save a list of your equianalgesic conversions. These data evidence your prescribing for re-validation purposes. Additionally, SPOT stores information for research and audit.

To access your list of previous conversions, select 'Previi-

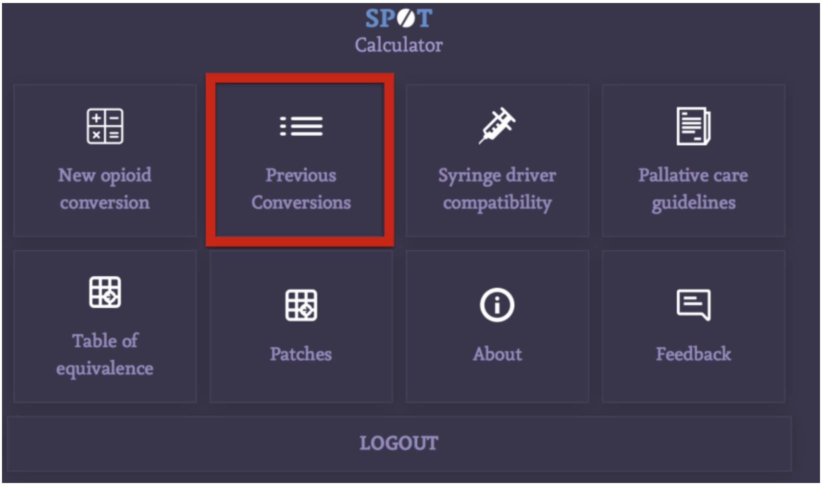

ous conversions' on the 'Home Screen'.

Your previous conversions are listed on the page:
You can select whether to see ‘All’ conversions, or “This

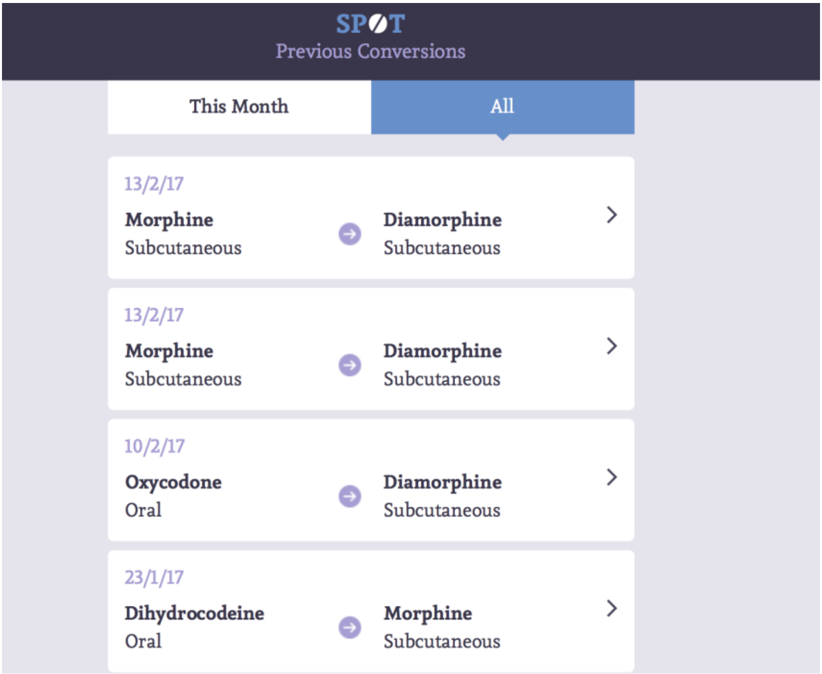

Month’s’.

Should you wish to see more detail on a particular con-version, click on the record. The date of the calculation is recorded for ease of reference.

Clicking on a record gives you information about the conversion. The patient's demographics are displayed, as well as the result of the calculation and if it did or did not

match. Additionally, there is the option to see the calcula-
tion

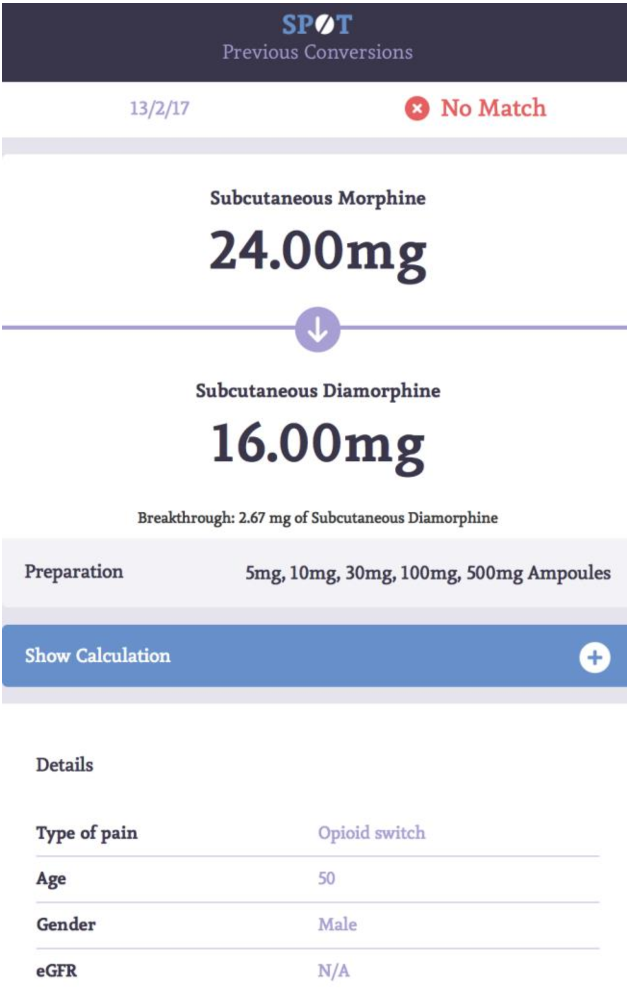

again by clicking ‘Show calculation’

Select the ‘Back button’ (highlighted in a red box below) in the top left hand side of the page to return to the

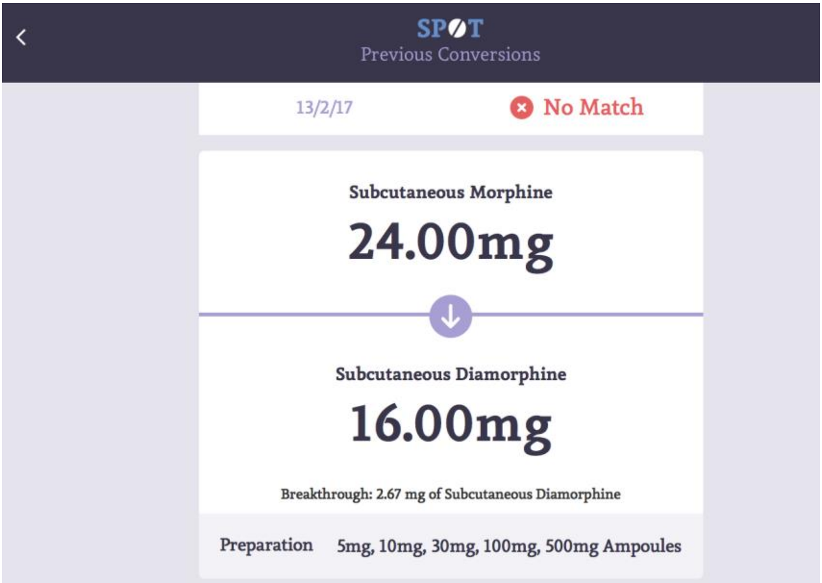

‘Home Screen’.

Chapter 8: Syringe Driver Compatibility and Scottish Palliative Care Guidelines

SPOT is designed to educate and inform. It provides links to the Scottish Palliative Care Guidelines Home Page and a direct link to the Syringe Driver Compatibility

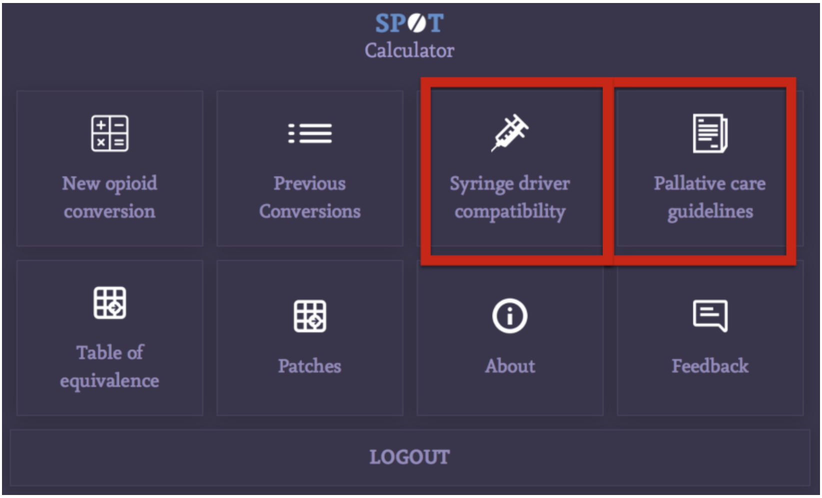

pages.

To access these resources from the ‘Home Screen’, ensure you have an active internet connection and click on the appropriate link. These will open in a new window. Close the window when you have finished to return to the SPOT ‘Home Screen’.

Chapter 9:Table of Equivalence and Patches

To review the equianalgesic tables and patch tables, se-

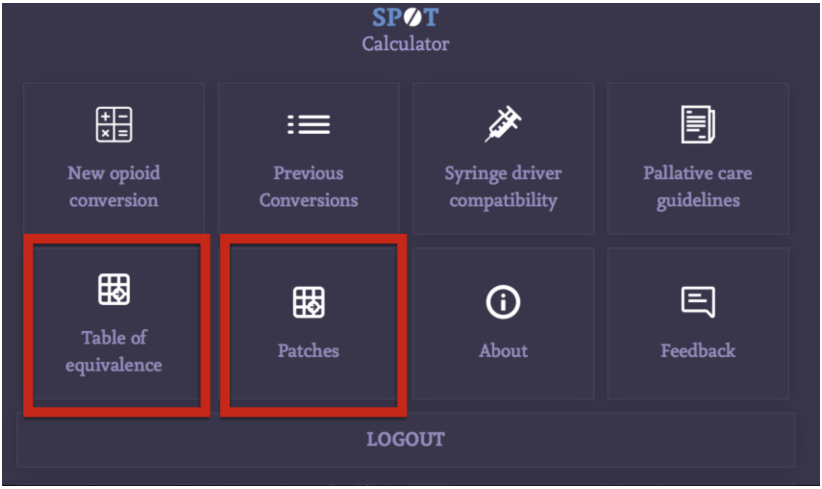

lect these from the ‘Home Screen’.

These tables form the basis of SPOT’s conversion algorithm and are based on the Scottish Palliative Care Guidelines.

The table of equivalence gives the equianalgesic ratio between the selected drug and route of administration, com-

SPOT
Table of Equivalence

Codeine

Admin route	Oral Morphine ratio
Oral	**0.1**

Dihydrocodeine

Admin route	Oral Morphine ratio
Oral	**0.1**

Morphine

Admin route	Oral Morphine ratio
Subcutaneous	**2.0**
Intravenous	**2.0**
Oral	**1.0**

Diamorphine

Admin route	Oral Morphine ratio
Subcutaneous	**3.0**
Intravenous	**3.0**

pared with 1mg of oral morphine. For example, 1mg of oral morphine is equivalent to 10mg oral codeine, or 0.5 mg subcutaneous morphine.

The patch table gives an approximate equianalgesic equivalence for transdermal fentanyl and Buprenorphine

Transdermal Fentanyl conversions

Oral morphine 24 hour intake (mg)	Fentanyl Patch equivalent (microgram per hour)
30	12
60	25
90	37
120	50
180	75
240	100

Transdermal Buprenorphine conversions

Oral morphine 24 hour intake (mg)	Buprenorphine Patch equivalent (microgram per hour)
12	5
24	10
48	20
84	35
126	52
168	70

conversions

To return to the home screen, select the ‘Back’ button in the top left hand corner.

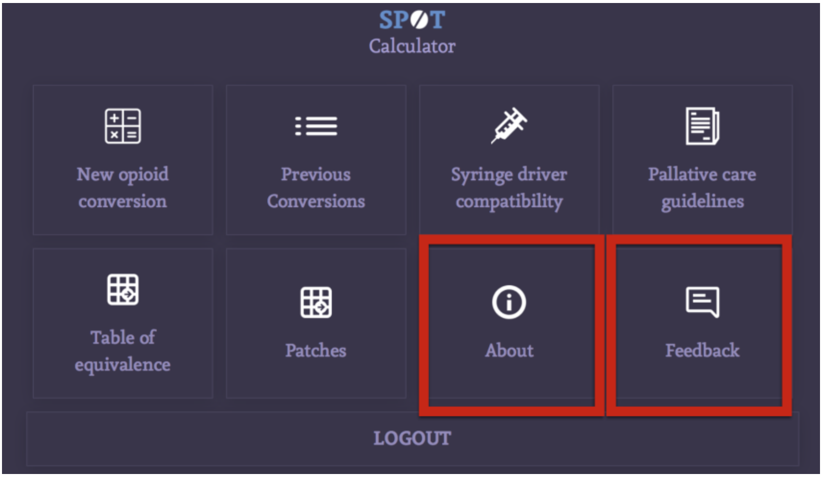

Chapter 10: About and Feedback
The 'About' page on the home screen links to
www.doctorflint.co.uk
Which tells you more about SPOT, its design and creation, and the team behind SPOT.

'Feedback" Sends a email directly to the SPOT team.

Chapter 11: Logout

Once you have finished using SPOT, remember to Logout.

Thank you for reading the instructions for use.

ENDS
