# Supplementary material for: The Safer Prescription of Opioids Tool (SPOT): A Novel Clinical Decision Support Digital Health Platform for Opioid Conversion in Palliative and End of Life Care—A Single-Centre Pilot Study
Source: Int J Environ Res Public Health. 2019 May 31;16(11):1926. doi: 10.3390/ijerph16111926 (PMC6612362; doi:10.3390/ijerph16111926)
Supplement: Supplementary file 1 [file ijerph-16-01926-s001.zip › ijerph-493995 Suppmentary Materials/ijerph-493995 Suppmentary 2.pdf]

Supplementary Material 2: SPOT YouTube video link

(updated for current iteration)
https://www.youtube.com/watch?v=AkbtwiUj4tc
https://www.youtube.com/watch?v=EF3a8SGit-o
https://www.youtube.com/watch?v=ER3jMpEWFnk&t=7s
